# Viewpoint: effective stakeholder communication in agriculture: together we stand, divided we fall!

**DOI:** 10.1017/S0021859614000276

**Published:** 2014-05-01

**Authors:** H. F. M. AARTS, J. HUMPHREYS, A. LE GALL

**Affiliations:** 1 Wageningen-UR (Plant Research International), P.O Box 616, 6700 AP Wageningen, Netherlands; 2 Teagasc, Moorepark, Fermoy, Co. Cork, Ireland; 3 Institut de l'Elevage, Paris, France

## Abstract

Substantial improvements of agricultural systems are necessary to meet the future requirements of humanity. However, current agricultural knowledge and information systems are generally not well suited to meet the necessary improvements in productivity and sustainability. For more effective application of research output, research producers and research consumers should not be considered as separate individuals in the knowledge chain but as collaborating partners creating synergy. The current paper investigates the relationships between scientists and stakeholders and identifies approaches to increase the effectiveness of their communication. On-farm research has proven to be an effective means of improving exploitation of research output at farm level because it connects all relevant partners in the process. Furthermore, pilot farms can act as an effective platform for communication and dissemination. Regional networks of pilot farms should be established and connected across regions.

## INTRODUCTION

Research has helped to understand and thereby to improve the functioning of agricultural systems. However, these systems have to be further improved to meet the food demands of a growing world population that is expected to grow from 7 billion in 2011 to 9 billion in 2050 (Godfray 2014). An average annual increase of productivity per unit area of 1% would suffice to meet this growing demand, but achieving this rate of growth is at risk (van Grinsven *et al.* 2014). For instance, the annual increase of wheat productivity was over 3% in 1970, but declined to just over 1% globally in 2010 and is currently <1% in Europe and the USA. (Dixon *et al.* 2009). In addition, more efficient utilization of energy, water and fertilizers is necessary because of limited resources and the need to reduce the environmental impacts of agriculture.

Better information and knowledge is needed by farmers, farming-related industries and governments at a time when European research budgets are being cut in most countries as a consequence of the European economic crisis from 2008 to 2014. Hence, research has to become more efficient and deliver more exploitable output per unit of investment. The European Commission recently concluded that there is a significant lag time between research output and implementation at farm level (European Commission 2012). At the same time the needs of practical farming are not being communicated effectively to the scientific community. Research results and expertise are poorly exploited and innovative ideas from practice are often not being captured, evaluated and verified. At the present time in Europe, the Agricultural Knowledge and Information Systems (AKISs) are generally not well suited to meet the necessary improvements in productivity and sustainability (EU SCAR 2012). This raises the question: how can the cooperation between scientists and their stakeholders be improved, resulting in more exploitable output from research?

## THE RELATIONSHIPS BETWEEN SCIENTISTS AND THEIR STAKEHOLDERS

From the scientists’ perspective, a stakeholder is someone that provides resources (inputs) to do research or that absorbs research results (outputs). Resource providers typically represent the people that consume the fruits of scientific work as a means of improving the effectiveness of their activities. While in the short term it can be profitable for a provider to cut investment in research to lower costs, in the longer term it can damage further business development. For a scientist, it can be interesting in the short term to neglect the actual knowledge needs of the resource provider, for instance by focussing on the knowledge gaps that are thought to be more exciting from a scientific point of view. However, when a scientist neglects the needs of the resource provider for too long or too often the provider will stop funding future work. Hence, researchers and their stakeholders are interdependent: if one fails, the other will also fail, perhaps after a period of increased happiness. Cooperation is essential for sustainable functioning of both.

Researchers fall roughly into three categories depending on their habitat: (1) universities or public research institutes for basic research, (2) applied research and development organizations and (3) research departments of commercial companies. There are also different categories of stakeholders: (1) farmers and farming industries, (2) government departments and agencies and (3) scientific journals. They all have their specific needs, history and thereby culture.

Farmers and farming industries want to implement knowledge and innovations to optimize their enterprise in order to stay in business. Generally this leads to a better utilization of resources, including land and labour. Governments also want to optimize farming practices and farming industries because it leads to higher employment, greater direct and indirect taxes due to increased productivity and results in better welfare and food security for the general voting public. In addition, governments develop and implement legislation to sustain or improve the quality of the environment through the reduction of the impacts of industries such as agriculture. Appropriate knowledge is needed to ensure that the legislation implemented is effective, balanced and targeted. Farmers (unions), farming industries and governments supply money for research and sometimes also research facilities, such as experimental farms, or provide means of dissemination and communication such as newspapers, magazines or websites, which can be very effective means of disseminating research results.

Scientific journals are quite different from the other stakeholders. They are interested in originalities in science and tend to be less interested in the actual applicability of new science in agricultural production systems. Originality of contributions is essential but it becomes more and more difficult to properly check offered papers, which often describe very detailed research. Commonly two or three reviewers are asked by the editor to give their opinions about originality and quality. The editor has to decide whether the paper should be published or not based on this advice. It is often difficult to find suitably qualified experts to provide an adequate review. Hence, to a certain extent, chance can play an important role in the acceptance of an offered manuscript. Journals provide the possibility to disseminate research results with the scientific community as the main target group. For academic researchers the number of published refereed publications is an important indicator for success. In that way the journals provide possibilities for the scientist to increase his scientific status and underpin opportunities for acquisition of new funding because the number of published scientific papers is often a criterion in the selection of project proposals. This stimulates the scientist to regard the scientific paper as the most important objective of his work rather than the applicability or impact of the research output.

## OBSTACLES TO THE EFFECTIVENESS OF RESEARCH

Governments require knowledge, mainly for legislation purposes and to help farmers and farming industry to solve problems and to stay competitive. But the current policy makers are often from non-agricultural or scientific backgrounds, which may compromise the decisions they make. Additionally, policy makers may temporarily work in governments/directorates, switching areas every 3 to 4 years. Communication with scientists is often by advertising funding for specified research and, in return, by written reports. Face-to-face communication is often infrequent and often not very effective because of differences in culture, interests and background knowledge. Furthermore, there is often a rather long period between the call for research and the delivery of resulting information by scientists. In that period, the political and economic situation may have changed considerably making the new information less relevant. Utilization of the information and knowledge delivered by scientists can also be poor because political and not scientific arguments may be an important consideration. These uncertainties in the use of outputs by governments, or absence of feedback from governments, stimulate scientists to focus on scientific journals.

Scientists working in applied research organizations and in research departments of commercial companies have the shared experience that there is intensive personal communication between the researcher and the user of his output, such as farm advisory services, farmers’ unions and farmers’ cooperatives. Unlike policy makers who regularly change areas, people employed in scientific organizations mostly stay within that organization for their entire working lives. In general, applied research scientists are rather familiar with culture and knowledge needs of their stakeholders and are often also involved in implementing knowledge, for instance by presentations to farmers or through on-farm demonstrations. In general, discovering how ‘old’ knowledge can contribute positively to the development of a farming system is more relevant than implementing novelties. For these scientists it can be difficult to publish in scientific journals, because there is the risk that reviewers will conclude that there is ‘nothing new’ in a synthesis of existing knowledge and its implementation at farm scale. Their main outputs are reports, articles in farmer's magazines and oral presentations for an audience of stakeholders. Traditionally, this job is mostly executed by people with family roots in agriculture. The number of scientists with such a background is declining. Moreover, it needs a number of rather non-productive years to learn how to do this integrating job properly. Nowadays, it is expected that scientists become productive immediately in terms of scientific publications; there is no time allowed to gain experience. In that case it is much more attractive to focus on detailed research, only considering a single component of the production system. This approach, however, risks shifting problems from one part of the farming system to another or replacing one environmental impact with another (Nemecek *et al.* 2011; Fenton *et al.* 2014).

This focus on detailed research is contrary to the growing need for a whole farm system research. Farming is becoming more and more complex and multifunctional; the production of food has to be combined with provision of other environmental goods and services, animal welfare, health and safety.

## IMPROVING RESEARCH EFFECTIVENESS THROUGH AN ON-FARM APPROACH

For improving the effectiveness of research, the producers and consumers of information and knowledge should not be seen as separate parts of the knowledge chain but as collaborating partners in the AKIS, with the intention of creating synergy (Röling 1990). Research on experimental and pilot farms offers opportunities for direct contact between farmers, farm advisors, people from the farming industry and researchers (Verloop 2013). Furthermore, it can combine research with effective knowledge dissemination, because farms can be visited and pilot farmers can present results, for instance, during meetings of farmers’ unions or discussion groups. On-farm research is an attractive way to realize the necessary improvement of the interconnectedness of AKIS. The effectiveness of this approach can be demonstrated by the results of the Dutch project ‘Cows & Opportunities’ and the Northwest-European project ‘DAIRYMAN’.

In the Dutch project Cows & Opportunities, scientists, farm advisers, people from dairy industries and dairy farmers have been collaborating intensively since 1998 (Oenema 2013). The objective of this long-term project is a cost-effective improvement of the environmental performances of dairy farms through optimizing resource management. Farm resource management adaptations that might be attractive are investigated, discussed by all, selected and tested on whole-farm scale on 16 pilot farms. These farms are distributed all over the Netherlands and represent all the main farming circumstances including, for example, soil type and hydrology. Farms are open for visits from other farmers and pilot farmers play an important role in knowledge dispersal. Farmer-to-farmer communication has proven to be most effective means of dissemination. Motivation and aptitude to communicate successfully were important criteria in the selection procedure for the 120 farmers that originally expressed the desire to be a pilot farmer. Experiences and results are not only used by farmers and farming industries to improve their businesses, but also by the Dutch government to define environmental regulations following consultation with the pilot farmers and scientists. Furthermore, the project network of people and pilot farms communicated agricultural and environmental regulations to the dairy sector. The project is on-going and the willingness to continue and the setting of new targets are discussed every 4 years. The Dutch government finances half of the project costs (€1·2 million/year); dairy farmers and milk processors finance the other half. In 2009 the government asked an independent organization to study the opinions of farmers and farm advisors as ‘knowledge consumers’ about the benefits emanating from the project (Zwart 2009). A telephone survey was conducted with 304 farmers and 24 farm advisors, representing the Dutch dairy sector. The results show that almost all Dutch farmers and farm advisors are familiar with the project (Table 1). Most of them made use of project output to improve their businesses. Many visited a pilot farm and feel that continuing on-farm research is important for progress in the development of their farm. Based on the results of Zwart's study (Zwart 2009), the Dutch government decided to continue the project. The ‘Cows & Opportunities’ approach now is called ‘the golden triangle approach’, expressing the benefits of a close cooperation between the agricultural sector, the government and scientists. To illustrate the benefits of this intensive collaboration the nutrient use efficiencies of the pilot farms was compared with those of non-pilot farms. The non-pilot farms (217 specialized dairy farms of the Dutch Farm Accountancy Data Network (FADN)) were selected to be representative of the average Dutch dairy farm. In both pilot farms and non-pilot farms, the farm inputs (imported feed and fertilizer) and farm outputs (milk and meat) were recorded and nutrient contents analysed. Nutrient use efficiency was calculated as outputs divided by inputs (Oenema *et al.* 2012).

**Table 1.** The awareness of farmers and farm advisors (‘knowledge consumers’) about the on-farm research project Cows & Opportunities and their thoughts about its practical value (Zwart 2009)

Survey statement	Farmers (*n*=304)	Farm advisors (*n*=24)
I know the project	96%	100%
I discussed the project with colleagues	39%	Not asked
I visited a pilot farm	32%	63%
I made use of information or tools, delivered by the project	65%	79%
In my opinion pilot farms are very important to test and demonstrate farm improvements	81%	100%
In my opinion the project should be continued	Not asked	92%

At the start of the project the nutrient use efficiencies of the pilot farms were considerably higher for N and slightly higher for P than those of the non-pilot farms (Table 2). This is not surprising, since those farmers initially volunteering to be a pilot farmer had an above average interest in improving their farm management. During the project, nutrient use efficiencies increased considerably across all farms. On the pilot farms N and P use efficiency increased by 13 and 44%, respectively, whereas the observed increases of N and P use efficiency on the non-pilot farms were 10 and 21%, respectively (Table 2). Altered management practices did not negatively affect the yields and quality of the crops or the milk production and health of the cattle. As a consequence, a pilot farmer now needs substantially less feed and fertilizer imported onto his farm per unit of milk production, which has reduced costs and increased farm income. This greater improvement in nutrient efficiency, by improved resource management, was stimulated by involvement in the project. The increase of nutrient use efficiency of non-pilot farmers was stimulated by stricter environmental regulations (for instance, a ban on surface spreading of manure and limits on fertilization periods) in combination with effective communication of the effectiveness and profitability of the new farm management practices demonstrated by the pilot farms in the Cows & Opportunities project.

**Table 2.** Average annual nutrient use efficiencies, defined as nutrients in farm outputs divided by nutrients in farm inputs (after Oenema 2013)

	Nitrogen		Phosphorus	
	1998	2011	1998	2011
Pilot farms	0·25	0·38	0·41	0·85
Non-pilot farms	0·20	0·30	0·39	0·60

The DAIRYMAN project used a similar methodology to the Cows & Opportunities project, but it extended over ten regions of Northwest Europe. Dairy farming is an important economic activity in these regions, which cooperated intensively during the project. Possibilities for more efficient utilization of feed and fertilizer (‘more with less’) were tested and demonstrated in a network of 131 pilot farms, which included the 16 farms of Cows & Opportunities project. A partnership approach between farm advisors, dairy industry representatives and farmers’ unions was taken to testing and demonstration of actions to improve farm management. The project included visits of farmers, farm advisors and scientists to their colleagues in other regions, interregional exchange of tools already used by farmers, cooperation in testing of innovations emerging from research and meetings with people from government and other stakeholders of rural areas to discuss needs and opportunities for improvement of sustainability (Aarts 2012). At the end of the project a survey was conducted to determine the opinions of people involved in the execution of the project, about failures and successes, which might prove to be useful in the design of similar projects. A survey form was sent to 120 ‘knowledge suppliers’ that were involved in the project, mainly scientists, of which 72 completed forms were returned. The results give an impression of their opinions about the value of this way of working (Table 3).

**Table 3.** The opinions of involved ‘knowledge suppliers’ (mainly scientists) about the on-farm research project DAIRYMAN (*n*=72)

Question	Score
Appreciation of working on the project (1=not at all, 10=very much)	%
Below 6	3
6 or 7	19
Above 7	78
Personal benefits (1=not at all, 5=very much)	Median
I gained new knowledge and skills	4
I enjoyed working with many different partners	4
I expanded my network	4
I learned from working in an international context	4
My work was meaningful	4
Presented striking examples (pilot farms) show that innovation works	%
Not at all	0
A little	9
Moderately	24
Quite a lot	49
Very much	18
There was good interaction between researchers, advisors and farmers	%
Not at all	0
A little	3
Moderately	18
Quite a lot	44
Very much	35

It can be concluded that ‘knowledge suppliers’ like this way of working, probably because they are able to expand their personal networks, to have direct contact with farmers and advisors, to increase their skills and because they notice that they really contribute to the improvement of farm practices. A quote: ‘*I found the DAIRYMAN project an excellent opportunity to make new contacts, to become aware of new technologies and to obtain a wider perspective on environmental issues by studying the situation in other dairy farming regions.’*

The position of pilot farmers is in between that of ‘knowledge consumers’ and ‘knowledge suppliers’. They consume knowledge, offered by scientists and farm advisors, to improve their farms. But they are also partners in testing of innovations and they demonstrate and explain progress in farm development to the farmers in their regions. From personal contact with the pilot farmers it is known that they really benefit from meetings with scientists, farm advisors and colleagues within and outside their region. A quote about the value of the farmers exchange programme of the project: ‘*It's an effective way to make people change their ways of thinking and working.*’ Almost all pilot farmers would join a follow-up project if the opportunity was presented to them.

## CONCLUSIONS

On-farm research has proven to be a beneficial means of to realising the necessary increase in the exploitability of research output because it connects all relevant partners of the AKIS. Pilot farms act as platforms where communication between science and farming practice is very effective. It can create what is really needed to bring about change at the farm level: a trust-based relationship, two-way communication and solid science. Therefore, to realize the desired increase in exploitable output of research, regional networks of pilot farms and the golden triangle of agricultural sector, the government and science should be established and connected across regions.
